# Ceramide Pathway Regulators Predict Clinical Prognostic Risk and Affect the Tumor Immune Microenvironment in Lung Adenocarcinoma

**DOI:** 10.3389/fonc.2020.562574

**Published:** 2020-10-27

**Authors:** Yuan Zhang, Jianbo Chen, Yunan Zhao, Lihong Weng, Yiquan Xu

**Affiliations:** ^1^Department of Pulmonary and Critical Care Medicine, Zhongshan Hospital, Xiamen University, Xiamen, China; ^2^The First Affiliated Hospital of Xiamen University, Xiamen, China; ^3^Department of Medical Oncology, Xiamen Key Laboratory of Antitumor Drug Transformation Research, The First Affiliated Hospital of Xiamen University, School of Clinical Medicine, Fujian Medical University, Xiamen, China; ^4^Shengli Clinical Medical College of Fujian Medical University, Fuzhou, China

**Keywords:** lung adenocarcinoma, prognostic risk signature, tumor immune microenvironment, consensus clustering, ceramide pathway

## Abstract

**Purpose:**

The ceramide pathway is strongly associated with the regulation of tumor proliferation, differentiation, senescence, and apoptosis. This study aimed to explore the gene signatures, prognostic value, and immune-related effects of ceramide-regulated genes in lung adenocarcinoma (LUAD).

**Methods:**

Public datasets of LUAD from The Cancer Genome Atlas and Gene Expression Omnibus were selected. Consensus clustering was adopted to classify LUAD patients, and a least absolute shrinkage and selection operator (LASSO) regression model was employed to develop a prognostic risk signature. CIBERSORT algorithm was used to estimate the association between the risk signature and the tumor immune microenvironment.

**Results:**

Most of the 22 ceramide-regulated genes were differentially expressed between LUAD and normal samples. LUAD patients were classified into two subgroups (cluster 1 and 2) and cluster 2 was associated with a poor prognosis. Furthermore, a prognostic risk signature was developed based on the three ceramide-regulated genes, Cytochrome C (CYCS), V-rel reticuloendotheliosis viral oncogene homolog A (RELA) and Fas-associated via death domain (FADD). LUAD patients with low- and high-risk scores differed concerning the subtypes of tumor−infiltrating immune cells. A moderate to weak correlation was observed between the risk score and tumor−infiltrating immune cells.

**Conclusions:**

Ceramide-regulated genes could predict clinical prognostic risk and affect the tumor immune microenvironment in LUAD.

## Introduction

Lung cancer is one of the most lethal cancers globally; in 2018, nearly 2.1 million new cases were diagnosed and 1.8 million deaths were recorded (1). In China, the morbidity of lung cancer ranks first and second among male and female patients, respectively, and has the highest mortality rate for both men and women (2). Lung adenocarcinoma (LUAD), a category of non-small cell lung cancer (NSCLC), is the most common histological type among all lung cancers (3). Clinically, more than 60% of patients with LUAD who show gene alterations at the time of diagnosis have an improved survival rate (4). Although developments in novel molecular-targeted drugs (5) and immune checkpoint inhibitors (6) have revolutionized the treatment of LUAD; treatment outcomes have only slightly improved, and the 5-year survival rate is only 15.6% (7). Furthermore, the high rates of drug resistance, recurrence, and metastasis are considered to be the main cause of the high mortality of this disease (8). Therefore, there is an urgent need to uncover more specific biomarkers of LUAD to develop effective diagnostic and therapeutic strategies.

Recently, the ceramide pathway, a core component of the sphingolipid metabolic pathway, has been widely studied in terms of its role in the regulation of tumor proliferation, differentiation, senescence, and apoptosis (9, 10). Ceramide is a membrane lipid that is synthesized by the *de novo* synthesis pathway of the endoplasmic reticulum or by the salvage pathway of lysosomes (11). Ceramide plays an important role in regulating membrane fluidity and subdomains, and can be produced in response to stress stimuli such as tumor necrosis factor-α (TNF-α), ionizing radiation, and chemotherapeutic drugs (12). Studies have shown that chemotherapy can induce the formation of ceramide; the inhibition of ceramide accumulation can lead cancer cells to become resistant to these stress stimuli (13, 14). Therefore, ceramide may play a role in mediating cell death by inducing apoptosis, and could play a key role in the development of resistance to chemotherapy. In fact, previous studies have shown that ceramide play key roles in a variety of cancers (15, 16), including lung cancer (17). In terms of LUAD, a previous study showed that ceramide promoted apoptosis in A549 human LUAD cells through the c-Jun N-terminal kinase (JNK) pathway (18). Although these findings have raised great interest in the possibility of treating cancer with ceramide, the mechanism by which ceramide perform their tumor-related role is not fully understood.

The development of novel approaches to cancer treatment, especially immunotherapy, brings new prospects to patients with lung cancer (19). Ceramide plays an important role in regulating the immune homeostasis and it was reported to participate in tumorigenesis and tumor progression. Ceramide exerts an inherent tumoricidal effect that blocks hepatocellular carcinoma (HCC) cell growth *in vivo* by inducing apoptosis *via* phosphorylation of the AKT pathway (20). Furthermore, ceramide was found to slow the growth of liver cancer *in vitro* by suppressing the anti-tumor immune response of tumor-associated macrophages and enhancing the anti-tumor effects of tumor antigen-specific CD8^+^ T cells (21). Ceramide is an important component of the T cell receptor (TCR) signaling mechanism and pharmacological or molecular inhibition of ceramide production can impact on TCR-induced interleukin-2 production and disrupt programmed cell death (22). A recent study showed that increased synthesis of ceramide results in deficiency of C-C motif chemokine receptor 5 and weakens CD4 + T cell memory response and antigen sensitivity (23). However, the anti-tumor immune mechanism of ceramide in LUAD remains unknown.

Thus, in this study, we analyzed the expression of these ceramide-regulated genes in LUAD patient samples and adjacent normal samples from both TCGA and Gene Expression Omnibus (GEO) datasets. In addition, we attempted to classify the cases into different subgroups based on differences in gene expression and prognosis, and to analyze the association between tumor-infiltrating immune cell types and different LUAD subgroups. We aimed to explore the different gene signatures, prognostic value, and impact on tumor immune microenvironment of ceramide-regulated genes in LUAD and explore the related therapeutic implications for LUAD treatment.

## Materials and Methods

### Data Collection

RNA-seq data of 594 study samples, including 535 LUAD biopsies and 59 healthy lung tissues, and the corresponding clinical information were obtained from the TCGA database (http://cancergenome.nih.gov/) (**Table 1**) as the training cohort in this study.

**Table 1:** Baseline characteristics of lung adenocarcinoma patients in the training cohort.

Variables	No. of patients	Percentage (%)
Total	522	100
Age (years)^*^		
≤ 60	160	30.65
> 60	343	65.71
Median (range)	66 (33–88)	
Gender		
Female	280	53.64
Male	242	46.36
Pathological stage^*^		
I	279	53.45
II	124	23.75
III	85	16.28
IV	26	4.98
T stage^*^		
T1	172	32.95
T2	281	53.83
T3	47	9.00
T4	19	3.64
N stage^*^		
N0	335	64.18
N1	98	18.77
N2	75	14.37
N3	2	0.38
M stage^*^		
M0	353	67.62
M1	25	4.79
Unknown	144	27.59

^*^Some patients had missing or uncertain data including 19 concerning age, 8 concerning pathological stage, 3 concerning T stage, 12 concerning N stage, and 144 concerning M stage.

Gene chip data from the public datasets of GEO (https://www.ncbi.nlm.nih.gov/geo/) were used as the validation cohort. Four GEO datasets, GSE30219, GSE32863, GSE37745, and GSE50081, which contained 448 samples, including 376 LUAD samples and 72 healthy control were selected.

Among the LUAD patients, 13 in the TCGA dataset and 2 in the GEO dataset had no clinical data and were excluded from analysis. This study was approved by the Medical Ethics Committee of Fujian Provincial Hospital (K2017-11-006).

### Identification of Differentially Expressed Genes

Limma package was used to identify differentially expressed genes between LUAD tissues and adjacent normal tissues. For gene expression data, reads per kilobase of transcripts per million mapped reads and the value expected to maximize normalization were obtained for the study samples.

### Gene Set Enrichment Analysis

Gene set enrichment and its potential functions for the ceramide pathway were evaluated *via* the Gene Set Enrichment Analysis (GSEA) website (http://software.broadinstitute.org/gsea/index.jsp) and BioCarta Pathway Database website (http://www.biocarta.com/genes/index.asp). We selected the hallmark gene set and a total of 45,956 genes and 50 gene sets were enrolled for GSEA. Gene sets with both false discovery rate (FDR) of <0.25 and normalized *p*-value of <0.05 were considered significant (24).

### Bioinformatic Analysis of Ceramide-Regulated Genes

The “ConsensusClusterPlus” algorithm (50 iterations, resample rate of 80%, and Pearson correlation, http://www.bioconductor.org/) in the R package for R v3.6.1 was used to cluster the LUAD samples into different groups according to the activity of ceramide-regulated genes. Principal component analysis (PCA) was used in the R package to analyze gene expression patterns in different LUAD groups. The STRING database (http://www.string-db.org/) was used to identify the interactions among ceramide-regulated genes.

### Construction of Prognostic Risk Score Model

Univariate Cox regression was performed to clarify the associations between the expression of ceramide-regulated genes and overall survival (OS). The LASSO Cox regression algorithm (25) was then used to develop an ideal prognostic model based on the expression of selected genes. In the LASSO Cox regression algorithm, the best penalty parameter λ and corresponding coefficients were determined by 10-fold cross-validation based on the minimum criteria. The risk score for the prediction model was calculated by the following formula:

$$Risk\;score\;=\;\sum_{i=1}^{n}Coefi\;*\;xi$$

Where, coef_i_ is the coefficient and x_i_ is the relative expression of the z-score transformation of each selected gene. This formula was applied to calculate the risk score for each patient in the TCGA database.

### Evaluation of Tumor−Infiltrating Immune Cell Types

The CIBERSORT algorithm (26) (1,000 permutations) was applied to characterize tumor-infiltrating immune cells in LUAD tissues, with the LM22 signature as a reference. LM22 is an annotated gene signature matrix that contains 547 marker genes to define 22 human immune cell subtypes, such as T cell types, B cell types, plasma cells, natural killer (NK) cells, and myeloid subpopulations. LM22 was downloaded from the CIBERSORT website (http://cibersort.stanford.edu/). We used 100 permutations from the default signature matrix and calculated the CIBERSORT p-value and root mean square error for each sample file to improve the accuracy of the deconvolution algorithm. Subsequently, we filtered and selected data from LUAD tissues with CIBERSORT values of *p <*0.05 for subsequent analysis. The immune cell components of LUAD samples from TCGA cohorts were analyzed using the CIBERSORT algorithm.

### TIMER Database

Information on tumor immune estimation was obtained from the TIMER database (https://cistrome.shinyapps.io/timer/), which comprises 10,897 samples across 32 cancer types from TCGA. Data on six types of immune infiltrates were collected, including B cells, CD4+ T cells, CD8+ T cells, neutrophils, macrophages, and dendritic cells.

### Human Protein Atlas

Differences in protein expression of CYCS, RELA, and FADD between human normal lung and LUAD tissues was revealed by immunohistochemistry (IHC) images from the Human Protein Atlas (HPA) website (https://www.proteinatlas.org). Selected antibodies were as follows: CYCS, CAB005126; RELA, CAB004264; and FADD, CAB010209. Protein expression score was automatically converted by the combination of staining intensity (negative, weak, moderate, or strong) and ratio of stained cells (< 25%, 25–75%, or > 75%), which was then classified into four levels as: 1) Not detect: staining negative or staining weak combined with ratio of stained cells <25%; 2) Low: staining weak combined with ratio of stained cells either 25–75% or 75%, or staining moderate combined with ratio of stained cells <25%; 3) Medium: staining moderate combined with ratio of stained cells either 25–75% or 75%, or staining strong combined with ratio of stained cells <25%; 4) High: staining strong combined with ratio of stained cells either 25–75% or 75%. In addition to this, protein expression values were manually adjusted as necessary when evaluated by expert annotators.

### Statistical Analysis

All statistical analyses were performed using R v3.6.0 and SPSS Statistics 20.0 (IBM, Inc., Armonk, NY, USA). Patients were divided into two groups (cluster 1 and cluster 2) based on the consensus expression of ceramide-regulated genes, or were classified into high- and low-risk groups according to the median risk score as a cut-off value. The Wilcox test was used to evaluate the difference in expression of ceramide-regulated genes in LUAD tissues and adjacent normal tissues, as well as the differences in the proportions of the 22 tumor-infiltrating immune cell subtypes in TCGA cohort. The Spearman test was used to identify correlations among ceramide-regulated genes and correlations between CYCS, RELA, and FADD expression and the abundance of immune infiltrates. The Chi-square test was performed to compare the distribution of age, gender, pathological stage, T stage, N stage, and M stage between the two groups. The Kruskal-Wallis or Wilcoxon test were conducted to compare the risk scores in patients grouped by clinical and pathological features. The Kaplan-Meier method with a two-sided log-rank test was adopted to compare the overall survival time between different groups. Uni- and multivariate Cox proportional hazard models were utilized to identify significant prognostic factors. A time-dependent receiver operating characteristic (ROC) curve was applied to evaluate the prediction efficiency of the prognostic risk score model. Values of *p <*0.05 were considered statistically significant.

## Results

### Differential Gene Expression in LUAD Tissues and Their Relationship Within Ceramide-Regulated Genes

**Figure 1:**
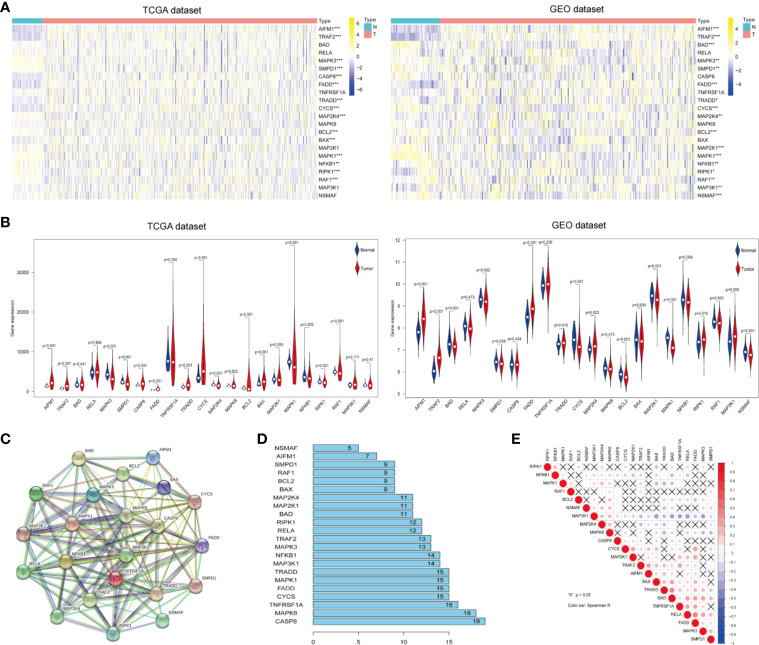
Differential expression of ceramide-regulated genes in lung adenocarcinoma (LUAD) tissues and their relationship with tumor progression. **(A, B)** Comparison of ceramide-regulated gene expression levels between tumor and normal tissues in TCGA and GEO datasets. **(C)** The modification-regulated interactions among ceramide-regulated genes in STRING database. **(D)** Number of interaction junctions among ceramide-regulated genes. **(E)** Spearman correlation analysis of the twenty-two ceramide-regulated genes. ^**^p < 0.01, ^***^p < 0.001.

To identify ceramide pathway-related genes and their potential functions, we searched the Gene Set Enrichment Analysis (GSEA) and BioCarta Pathway Database website, and found 22 genes including apoptosis-related genes such as RELA, CASP8, BAD, and BAX; inflammatory response genes such as TRAF2, RAF1, and NFKB1, and hypoxia-related genes such as BCL2 and MAP3K1 (**Supplementary Table S1**). To determine the essential biological functions of ceramide in tumorigenesis and tumor development, we analyzed the expression of these ceramide-regulated genes in LUAD patient samples and adjacent normal samples in both the training (TCGA) and validation (GEO) cohorts. The results showed that the expression of most ceramide-regulated genes was significantly different between LUAD and normal samples (**Figure 1A**). The expression of genes including TRADD, TRAF2, AIFM1, and FADD was significantly increased in LUAD samples (both *p* < 0.001) compared to that in normal tissues, and genes such as MAPK3 (*p* < 0.01), NFKB1 (*p* < 0.01), SMPD1 (*p* < 0.01), BCL2 (*p* < 0.001), MAPK1 (*p* < 0.001), and RAF1 (*p* < 0.01) showed significantly decreased expression (**Figure 1B**) in both the TCGA and GEO datasets.

Next, we attempted to elucidate the association between these ceramide-regulated genes. As shown in **Figures 1C, D**, CASP8 appeared to act as a hub gene for ceramide pathways, and interacted with 19 other genes according to the String database. However, the correlation analysis failed to show a strong correlation between CASP8 expression and the expression of other ceramide-related genes (**Figure 1E**). However, interestingly, FADD showed interactions with 15 other genes, and its expression was the most strongly positively correlated with that of RELA, followed by CYCS (**Figure 1E**). Altogether, the significantly higher expression of FADD in LUAD suggests that this gene may be a key regulator of the ceramide-related pathways.

### Consensus Clustering of Ceramide-Regulated Genes Identified Two Clusters for LUAD

**Figure 2:**
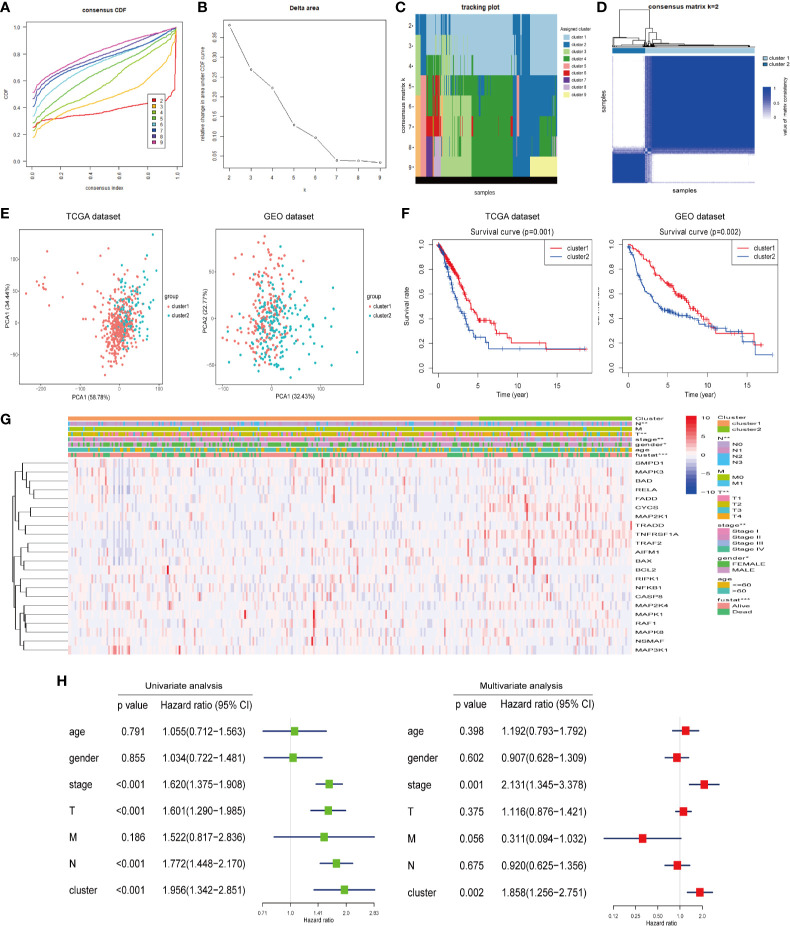
Differential clinicopathological features and overall survival (OS) of LUAD in the cluster 1/2 subgroups. **(A)** Consensus clustering cumulative distribution function (CDF) of k = 2-9 in TCGA dataset. **(B)** Relative change in the area under the CDF curve for k = 2-9 in the TCGA dataset. **(C)** Tracking plot for k = 2-10 in the TCGA dataset. Different consensus matrix k classifies the samples into corresponding number of clusters displayed in different colors. Each column represents a sample and each row represents a consensus matrix k-value, and each color represents a cluster that samples gets assigned to. **(D)** The consensus clustering matrix k = 2 divides the TCGA dataset into two clear distinct clusters. The rows and columns represent the samples and the value of the matrix consistency is represented by white to blue from 0 (impossible clustering together) to 1 (always clustering together). **(E)** Principal component analysis of expression profiles between cluster 1 and cluster 2. **(F)** Kaplan-Meier analysis of overall survival (OS) for cluster 1/2 subgroups. **(G)** The relationship between clinicopathologic features and the two clusters (cluster 1/2) as defined by the expression of ceramide-regulated genes. **(H)** Univariate and multivariate Cox proportional hazard regression analyses of the association between the risk score (including clinicopathological factors) and OS of LUAD patients from the TCGA dataset. ^*^p < 0.05, ^**^p < 0.01, ^***^p < 0.001.

We constructed consensus clusters of the LUAD patients in the training cohort based on the similarity of ceramide-regulated gene expression. According to the consensus clustering cumulative distribution function (CDF) (**Figure 2A**) and relative changes in the area under the CDF curve (**Figure 2B**), the stability of clustering increased continuously from consensus matrix k = 2 - 9 in these datasets. In addition, when taking the sample distribution of the considered k values, k = 2 was found to be the most accurate for consensus clustering, and the training cohort samples were divided into two clearly distinct clusters (**Figures 2C, D**). Thus, we classified the LUAD patients into two subgroups: cluster 1 and cluster 2, and the characteristics of the patients are listed in **Table 2**. To further verify the accuracy of our consensus clustering, we compared the transcriptional profiles of cluster 1 and cluster 2 by PCA. A relatively distinction was observed between them in TCGA and GEO datasets (**Figure 2E**). We then compared the difference in OS between these two subgroups, and the result uncovered that cluster 2 had a significantly lower OS than cluster 1 in the TCGA dataset (*p* = 0.001, left plot in **Figure 2F**). This result was further confirmed in GEO dataset (*p* = 0.002, right plot in **Figure 2F**). Moreover, we analyzed the differences in clinicopathological features between the two subgroups in the TCGA dataset. Cluster 2 was significantly associated with male patients (*p* < 0.05), a high pathological stage (*p* < 0.01), and malignant T stage (*p* < 0.01) and N stage (*p* < 0.01, **Figure 2G**). To verify potential confounding effects included by the clinical features, we performed a uni- and multi-variate analyses. The univariate Cox regression showed that the clustering was significantly associated with the OS of LUAD patients [hazard ratio (HR) = 1.956, 95% confidence interval (CI) = 1.342–2.851, *p* < 0.001, **Figure 2H**, left plot]. This association was still clear even after adjusting the multivariate Cox proportional hazard model for clinical parameters of the patients, including age, gender, and TNM stage (HR = 1.858, 95% CI = 1.256-2.751, *p* = 0.002, **Figure 2H**, right plot). Collectively, these findings indicated that the consensus clustering of ceramide-regulated genes may be independently associated with worse prognosis of LUAD patients.

**Table 2:** Clinical characteristics are different between cluster1 and cluster2.

Variables	Cluster1, N = 245 (%)	Cluster2, N = 91 (%)	p-value*
Age (years)			0.05
≤ 60	72 (29.39)	37 (40.66)	
> 60	173 (70.61)	54 (59.34)	
Median (range)	66 (33–87)	64 (38–85)	
Gender			0.016
Female	133 (54.29)	36 (39.56)	
Male	112 (45.71)	55 (60.44)	
Pathological stage			0.002
I	135 (55.10)	36 (39.56)	
II	59 (24.08)	23 (25.27)	
III	33 (13.47)	28 (30.77)	
IV	18 (7.35)	4 (4.40)	
T stage			0.004
T1	84 (34.29)	16 (17.58)	
T2	135 (55.10)	55 (60.44)	
T3	15 (6.12)	13 (14.29)	
T4	11 (4.49)	7 (7.69)	
N stage			0.007
N0	166 (67.76)	45 (49.45)	
N1	47 (19.18)	24 (26.37)	
N2	32 (13.06)	21 (23.08)	
N3	0 (0.00)	1 (1.10)	
M stage			0.331
M0	227 (92.65)	87 (95.60)	
M1	18 (7.35)	4 (4.40)	

*Chi-square test for distributions between cluster1 and cluster2.

### Identification and Validation of a Risk Signature Predicting Survival in LUAD Patients

**Figure 3:**
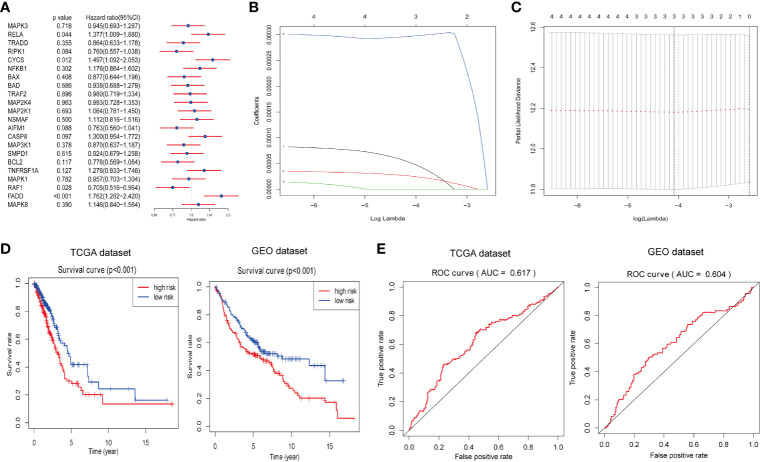
Construction of prognostic risk signature for LUAD patients with three ceramide-regulated genes. **(A)** Univariate Cox proportional hazard regression analysis of the association between expression levels of the indicated genes and overall survival of LUAD patients from the TCGA dataset. **(B)** LASSO Cox regression algorithm showing the coefficients of the selected genes in the TCGA dataset. **(C)** Tuning the penalty parameter in LASSO Cox regression using 10-fold cross validation in the TCGA dataset. **(D)** Kaplan-Meier overall survival curves for patients with LUAD based on risk signature. **(E)** The 3-year ROC curves showing the predictive accuracy of the risk signature for LUAD patients.

Next, we attempted to explore the prognostic role of ceramide-regulated genes in LUAD. Through univariate Cox proportional hazard regression analysis on the expression levels of these genes in the TCGA dataset, 4 out of 22 tested genes were found to be significantly associated with OS (*p* < 0.05, **Figure 3A**). Of the four genes identified, RELA, CYCS, and FADD were found to be associated with a high risk, showing a HR of >1, whereas RAF1 was identified as a low-risk gene, with HR <1. In order to more accurately predict the clinical prognosis of LUAD based on the expression of ceramide-regulated genes, we performed the LASSO Cox regression algorithm based on the above four prognosis-associated genes in TCGA dataset, which was used as the training cohort (**Figures 3B, C**). Three genes, RELA, CYCS, and FADD, were selected to establish the risk signature, and the coefficients used to calculate the risk score were determined after 10-fold cross-validation. The coefficients for RELA, CYCS, and FADD were found to be 5.0637, 2.5219, and 0.0003, respectively. Moreover, based on the risk signature, the LUAD patients were classified into low- and high- risk subgroups in both the training and validation datasets. As depicted in **Figure 3D**, patients with a high-risk score had a significantly lower OS than those with a low-risk score in both datasets (both *p* < 0.001). To investigate the predictive accuracy of this risk signature, we performed time-dependent ROC analysis. The area under the ROC curve (AUC) for the 3-year analyses in the training and validation datasets was 0.617 and 0.604, respectively (**Figure 3E**). These results indicated that this risk signature performed relatively well in predicting the survival of LUAD patients.

### Protein Expression of the Selected Risk Signature Genes, and the Relationship Between Clinicopathological Features and Prognostic Risk Scores

**Figure 4:**
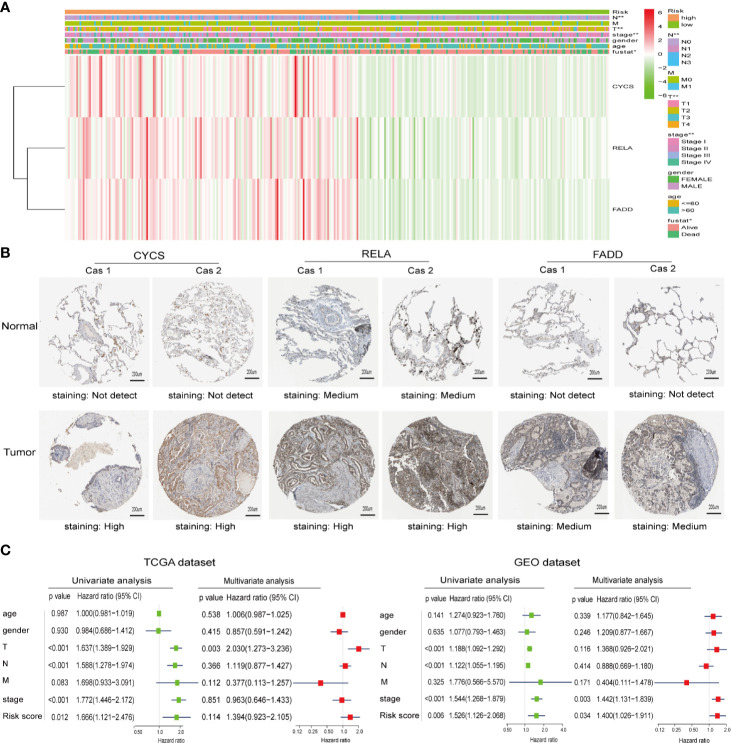
Protein expressions of the selected risk signature genes and the association between clinicopathological features and prognostic risk scores. **(A)** The distribution of clinicopathological features and the expression levels for selected risk signature genes in the ceramide pathway were compared between the low- and high-risk subgroups from the TCGA dataset. **(B)** The protein expressions of CYCS, RELA, and FADD in LUAD tissues and normal lung tissues by the Human Protein Atlas. **(C)** Uni- and multivariate Cox regression analyses of the relationship between the risk score (including clinicopathological factors) and overall survival of LUAD patients. ^*^p < 0.05, ^**^p < 0.01.

To investigate the relationship between clinicopathological features and risk scores, we collected clinicopathological data from patients with low- or high-risk scores and constructed a heatmap. As shown in **Figure 4A**, the expression of the three selected ceramide-regulated genes, CYCS, RELA, and FADD was increased in the high-risk subgroup. We also analyzed the relationship between clinicopathological features and the risk score, and identified significant differences in the risk score based on pathological stage (*p* = 0.002), T stage (*p* = 0.001), N stage (*p* = 0.002), and cluster 1/2 (*p* < 0.001) (**Figure 4A** and **Figure S1**). However, we were unable to find an association between the risk score and age, gender, or M stage. In addition, we attempted to determine the protein expression levels of CYCS, RELA, and FADD in LUAD using the HPA database. The IHC test showed that CYCS and FADD were not expressed in normal lung tissues, whereas high and medium expression levels of them, respectively, were observed in LUAD tissues. (**Figure 4B**). Moreover, RELA was highly expressed in LUAD tissues, whereas medium expression levels were observed in normal lung tissues. To detect whether the risk signature is an effective and independent prognostic indicator, we used uni- and multivariate Cox regression to analyze the LUAD dataset. The result of univariate Cox regression analysis showed that the risk score was significantly associated with poor patient outcome (HR = 1.666, 95% CI = 1.121–2.476, *p* = 0.012, **Figure 4C** left plot). However, the risk score lost its predictive potential in the TCGA dataset after adjusting for clinical pathological features of the patients (HR = 1.394, 95% CI = 0.923–2.105, *p* = 0.114). Despite of these contradictory results, assessment of the GEO dataset revealed that risk score was significantly associated with poor prognosis of LUAD patients in both the uni- and multi-variate Cox regression analyses (HR = 1.526, 95% CI = 1.126–2.066, *p* = 0.006 and HR = 1.400, 95% CI = 1.026–1.911, *p* = 0.034, respectively, **Figure 4C** right plot). These results indicated that the prognostic risk score derived from ceramide-regulated gene profile has the potential to predict prognosis of patients with LUAD.

### Relationship Between Tumor−Infiltrating Immune Cells, Patients With Different Risk Scores and Expressions of the Three Selected Risk Signature Genes

**Figure 5:**
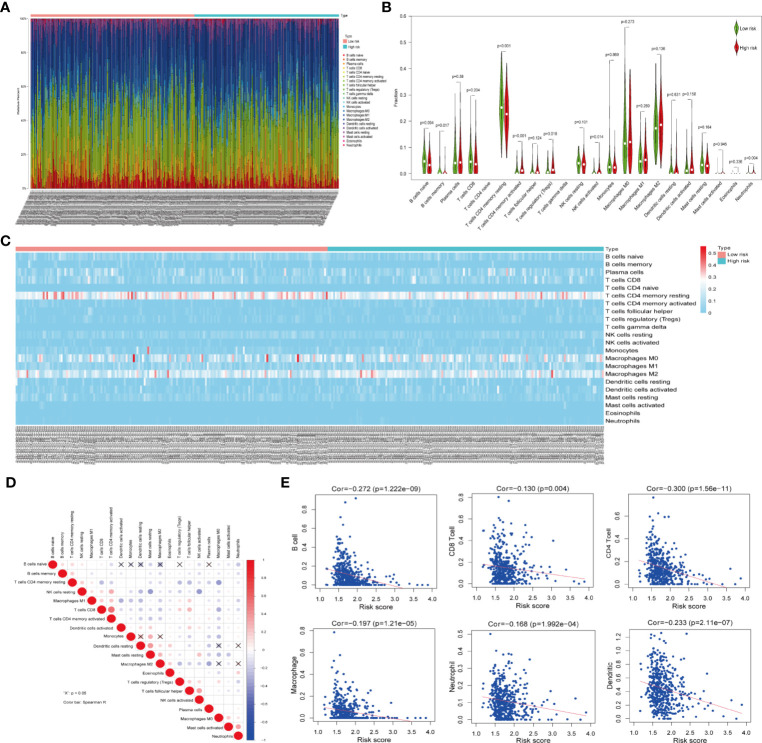
Relationship between tumor-infiltrating immune cells, patients with different risk scores and expressions of the three selected risk signature genes in TCGA dataset. **(A)** Composition of tumor-nfiltrating immune cells in two subgroups (low- and high-risk) of LUAD. **(B)** Distributions of tumor-infiltrating immune cell types between the low- and high-risk subgroups. **(C)** Heatmap showing the distribution of different tumor-infiltrating immune cell types among the two subgroups. **(D)** Spearman correlation analysis of the twenty tumor-infiltrating immune cells. **(E)** The correlation between the risk score and six tumor-infiltrating immune cell subtypes.

Owing to the important role of tumor-infiltrating immune cells in tumor development and its impact on prognosis, we analyzed their fractions in LUAD tissues based on the risk signature. By filtering with CIBERSORT for values of *p <*0.05, we found that CD4 naive T cells and gamma delta T cells were excluded as they were not expressed in either high- or low-risk subgroups (**Figure 5A**). Thus, a total of 20 tumor-infiltrating immune cells were used for analysis in 153 high-risk patients and 173 low-risk patients. CD4 memory resting T cells, M0 macrophages, and M2 macrophages were the most common tumor-infiltrating immune cells in both high- and low-risk patients (**Figures 5A–C**). Furthermore, three tumor-infiltrating immune cell subsets, including naïve B memory cells, memory B cells, and CD4 memory resting T cells were present in significantly higher fractions in high-risk patients as compared with low-risk patients (*p* < 0.05, **Figures 5B, C**). However, CD4 memory activated T cells, regulatory T cells, and activated NK cells and neutrophils were found at lower frequencies in high-risk patients than in low-risk patients (*p* < 0.05, **Figures 5B, C**). In addition, the relationship between each tumor-infiltrating immune cell type was analyzed. Among the tumor infiltrating immune cells, the CD4 memory activated T cells showed a strong and positive associated with CD8 T cells, whereas resting dendritic cells were moderately associated with resting mast cells (**Figure 5D**). Moreover, activated NK cells were positively associated with follicular helper T cells, whereas negatively associated with resting NK cells. Last, we investigated the relationship between risk score and tumor-infiltrating immune cells. The results revealed that the risk score was moderately correlated with all the CD4+T cells and weakly associated with other five tumor-infiltrating immune cell subtypes, including B cells, CD8+T cells, macrophages, neutrophils, and dendritic cells (both *p* < 0.05, **Figure 5E**). These findings indicated that the risk signature based on ceramide pathway regulators could classify the different profiles of tumor immune cells infiltrated in LUAD.

## Discussion

Its high recurrence rate and poor prognosis make LUAD one of the most lethal diseases worldwide (27). The ceramide pathway has been reported to play a vital role in the occurrence and development of several tumor types, including LUAD (17, 18). However, the mechanisms through which the ceramide pathway acts on LUAD remain unclear. In this study, we found that ceramide-regulated genes were highly expressed in LUAD, and two subgroups were identified, which showed a close association with clinicopathologic features and prognosis based on consensus clustering. We further constructed a prognostic risk signature that stratified the LUAD patients into high- and low-risk subgroups. In addition, we discovered that the composition of tumor-infiltrating immune cells in the two risk subgroups were different. To our knowledge, this is the first study to report that ceramide-regulated genes could predict clinical prognostic risk and affect the tumor immune microenvironment in LUAD.

Recent research has increasingly focused on whether ceramide-regulated genes can be used as a prognostic biomarker for cancer. Sabrina et al. (28) showed that ceramide-regulated gene expression is significantly reduced in HCC tissues compared to adjacent normal tissues, indicating that these genes may be biomarkers for HCC. Another study showed that ceramide-regulated gene expression is significantly increased in human breast cancer tissues and is associated with less aggressive cancer biology, indicating that the expression of ceramide-regulated genes may also be used to predict the prognosis of breast cancer (29). In our study, we constructed a prognostic risk signature derived from three ceramide-regulated genes; this risk signature was proven valuable. In addition, our risk signature could stratify OS in terms of pathological stage, T stage, and N stage. Our findings indicate that the prognostic risk signature based on the expression of ceramide-regulated genes has the potential to predict prognosis of LUAD patients. Although another prognosis predictive model for LUAD has been reported, the sample size was small (76 NSCLC samples vs. 75 normal samples) and the predicted value was relatively low (30). Additionally, it is undeniable that other commonly altered pathways enriched by the 22 identified genes, such as apoptosis pathways enriched by Bax, Bcl-2, and Bad, may be contribute to the risk signature and impact on the risk signature potential to predict prognosis independently. A better designed and independent prognostic model will need to be developed in the future.

In this study, we found that the ceramide pathway comprised 22 genes, and that CYCS, FADD, and RELA were more highly expressed in LUAD samples than that in normal tissues. These three ceramide-regulated genes were also selected to construct our prognostic risk signature. Thus, CYCS, FADD, and RELA show potential as key regulators of the ceramide pathway in LUAD.

CYCS is a mitochondrial protein that participates in the formation of apoptotic bodies by binding to apoptotic protease activator-1 (Apaf-1) and procaspase-9, and ultimately induces apoptosis when it is released into the cytoplasm (31). Additionally, CYCS was reported to be associated with ceramide, which interacts with BAX to change the permeability of mitochondrial membrane and promote the release of CYCS, ultimately leading to apoptosis (32). A previous study revealed that the nuclear localization of CYCS could be induced by evodiamine to stimulate apoptosis independent of the p53 pathway in lung cancer cells (33). In our study, CYCS expression was significantly increased in LUAD compared to that in normal tissues, and was associated with a poor OS. This indicates that CYCS may be an oncogene in LUAD. Furthermore, previous research has shown that serum CYCS levels are related to malignant progression and poor prognosis in NSCLC (34), consistent with our result. These findings indicate that CYCS may play different roles in different tumors, and the underlying molecular mechanisms require further study.

Similar to *CYCS*, *FADD* was highly expressed in LUAD, and was therefore selected to construct the prognostic risk signature in the present study. FADD is a critical adaptor protein that transmits apoptosis signals by interacting with cell-surface death receptors (DRs) and recruiting caspase-8. Recent studies have indicated that FADD expression is correlated with tumor development (35). FADD expression was previously found to be significantly increased in LUAD and was associated with a poor prognosis (36). A previous study revealed that patients with lymph node metastasis (LNM) in head and neck squamous cell carcinoma showed higher FADD expression than those without LNM (37). Furthermore, the loss of FADD was shown to lead to the inhibition of T cell proliferation and dysregulated cell cycle progression, suggesting that FADD plays a key role in these processes (38). In this study, increased FADD expression was detected in patients with LUAD and was correlated with a poor OS, consistent with the above previous findings. However, the molecular mechanisms leading to these effects of FADD on LUAD are unclear and require further investigation.

RELA, also known as p65, is one of the most common NF-κB subunits, and is involved in the classical NF-κB pathway (39). Previous research has demonstrated that RELA interacts with Alpha-actinin 4 (ACTN4) to induce proliferation and apoptosis in NSCLC cells (40). Furthermore, the nuclear overexpression of RELA was found to be significantly associated with a poor prognosis in patients with diffuse large B-cell lymphoma. In our study, high RELA expression was associated with a poor prognosis in patients with LUAD, and was also selected to build our prognostic risk signature. Based on our results and those of previous findings, RELA is associated with poor survival in several cancers and may be a key oncogene for tumorigenesis and development. In addition, the present findings in the above three genes may also help to develop new treatments by characterizing the expression of all ceramide-regulated genes in LUAD.

Recently, the tumor immune microenvironment has gained widespread attention in cancer research (41). To assess the fractions of immune cells in LUAD patients with different clusters or risk scores, we applied the CIBERSORT method, which can be used to analyze cellular and tumor specimens by microarray or RNA-Seq (26). Unlike immunohistochemistry, which is largely dependent on limited phenotypic characteristics and requires multiple parameters to accurately determine subpopulations, CIBERSORT is a newly developed metagene-based method that provides comprehensive immune cell subtype analysis for a large number of samples in a short period of time (42), and its effectiveness has been confirmed (26).

Previous studies confirmed the role of tumor-infiltrating immune cells in LUAD (43, 44). A comprehensive characterization of immune cells in LUAD showed that CD8+ T cells accumulation is associated with a poor prognosis (45). In addition, M2 macrophages have been found to promote lung cancer cell invasion and xenograft tumor growth, whereas M1 macrophages induce apoptosis and inhibit tumor formation (46). A previous study showed that ceramide reduced the proportion of M2 macrophages and increased that of CD8^+^ T cells to enhance the efficacy of immune therapy in HCC (21). In our study, six tumor-infiltrating immune cell subtypes were found to be present in different frequencies between the high- and low-risk subgroups, and the risk score was moderately correlated with the CD4^+^T cells and weakly associated with five other tumor-infiltrating immune cell subtupes. These findings indicate that ceramide pathway regulators could impact on tumor-infiltrating immune cells, in particular CD4^+^ T cells, and further regulate the tumor immune microenvironment in LUAD.

There are some limitations in our study that should be taken into consideration. First, although the risk signature performed relatively well in predicting the survival of LUAD patients, it is inevitably affected by clinicopathological factors. Second, the correlation between the risk score and the tumor-infiltrating immune cells ranged from moderately to weakly. Third, although the newly developed CIBERSORT method was used for immune cell analysis in the TCGA cohort, there was a lack of independent validation datasets such as animal models or human tissues to further confirm our findings. Therefore, well-designed and in-depth studies are warranted to overcome these limitations.

**Figure 6:**
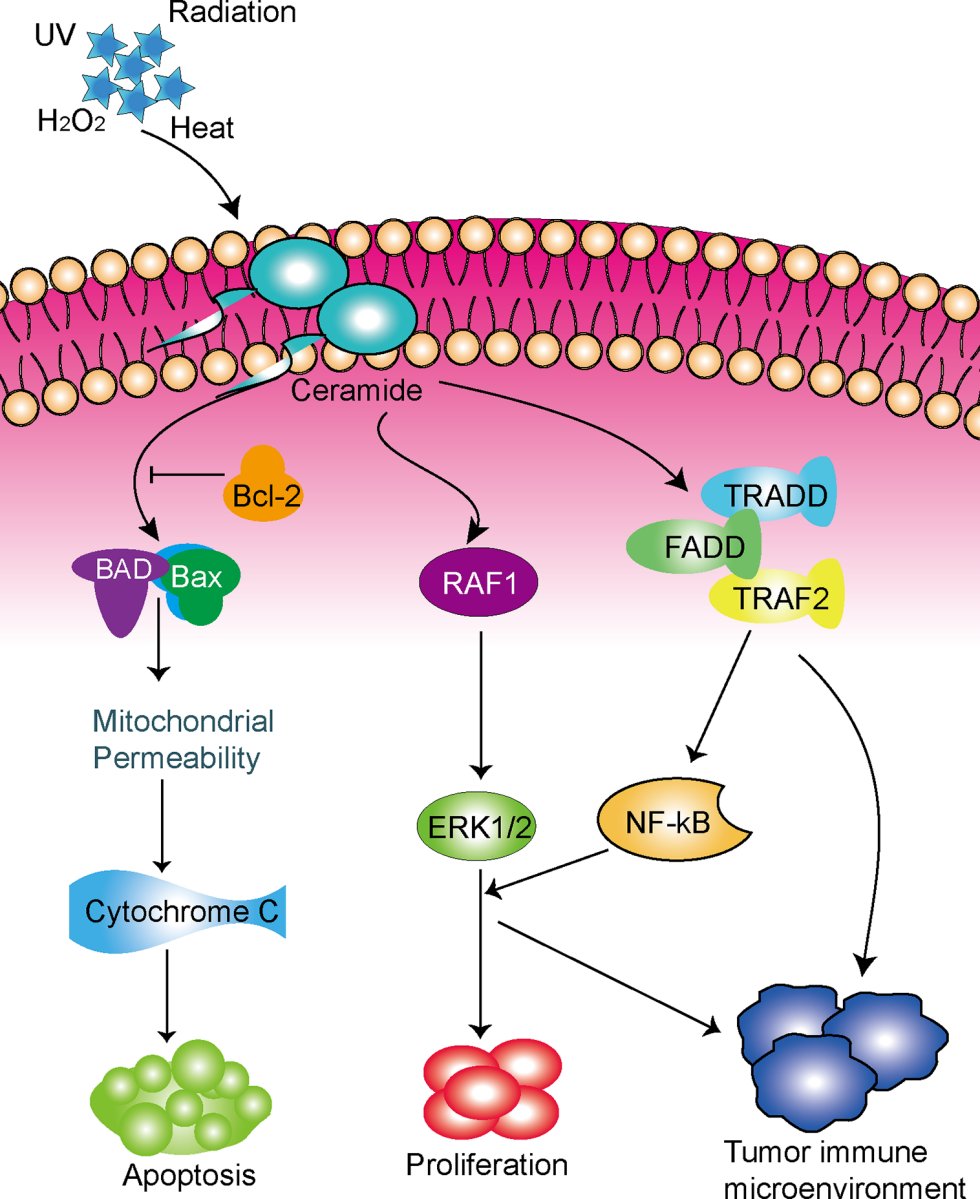
Schematic diagram of the potential functions of the ceramide pathway regulators in LUAD.

Taken together, the role of ceramide-regulated genes in LUAD has not been fully clarified, and further investigation is greatly needed. Meanwhile, our study demonstrated that ceramide-regulated genes expressed differently in LUAD tissues and normal lung tissues, and we identified two subgroups associated with malignant clinicopathologic features and poor prognosis. We also developed a prognostic risk signature and found that ceramide pathway regulators could affect the tumor immune microenvironment in LUAD (**Figure 6**). Hence, the ceramide-regulated genes identified here show promise as prognostic markers for LUAD, and may be helpful for predicting survival and selecting effective treatment strategies.

## Conclusion

In summary, Ceramide pathway regulators predict clinical prognostic risk and impact tumor immune microenvironment in LUAD, and may be a novel prognostic factor for LUAD patients.

## Data Availability Statement

The datasets presented in this study can be found in online repositories. The names of the repository/repositories and accession number(s) can be found in the article/**Supplementary Material**.

## Ethics Statement

The studies involving human participants were reviewed and approved by the Medical Ethics Committee of Fujian Provincial Hospital (K2017-11-006). The patients/participants provided their written informed consent to participate in this study.

## Author Contributions

YX designed and conducted the study and was responsible for writing the manuscript. JC, YZhao, and LW contributed to the data collection and analysis. YX, JC, YZhao, and YZhan crafted the literature search, figures, and tables. YZhan and YX contributed to the experimental design, the review, and revision of the manuscript. All authors contributed to the article and approved the submitted version.

## Funding

This study was funded by Startup Fund for scientific research, Fujian Medical University (Grant number: 2017XQ2046).

## Conflict of Interest

The authors declare that the research was conducted in the absence of any commercial or financial relationships that could be construed as a potential conflict of interest.

## Supplementary Material

Supplementary Figure 1 Association between the risk score, clinicopathological features, and cluster 1/2 subgroups in TCGA dataset. Distribution of risk scores in LUAD patients stratified by age **(A)**, gender **(B)**, pathological stage **(C)**, T stage **(D)**, N stage **(E)**, M stage **(F)** and cluster 1/2 subgroup **(G)**.

Supplementary Table 1 Gene list of ceramide pathway.
