# Supplementary figures and images for: Ceramide Pathway Regulators Predict Clinical Prognostic Risk and Affect the Tumor Immune Microenvironment in Lung Adenocarcinoma
Source: Front Oncol. 2020 Oct 27;10:562574. doi: 10.3389/fonc.2020.562574 (PMC7653182; doi:10.3389/fonc.2020.562574)

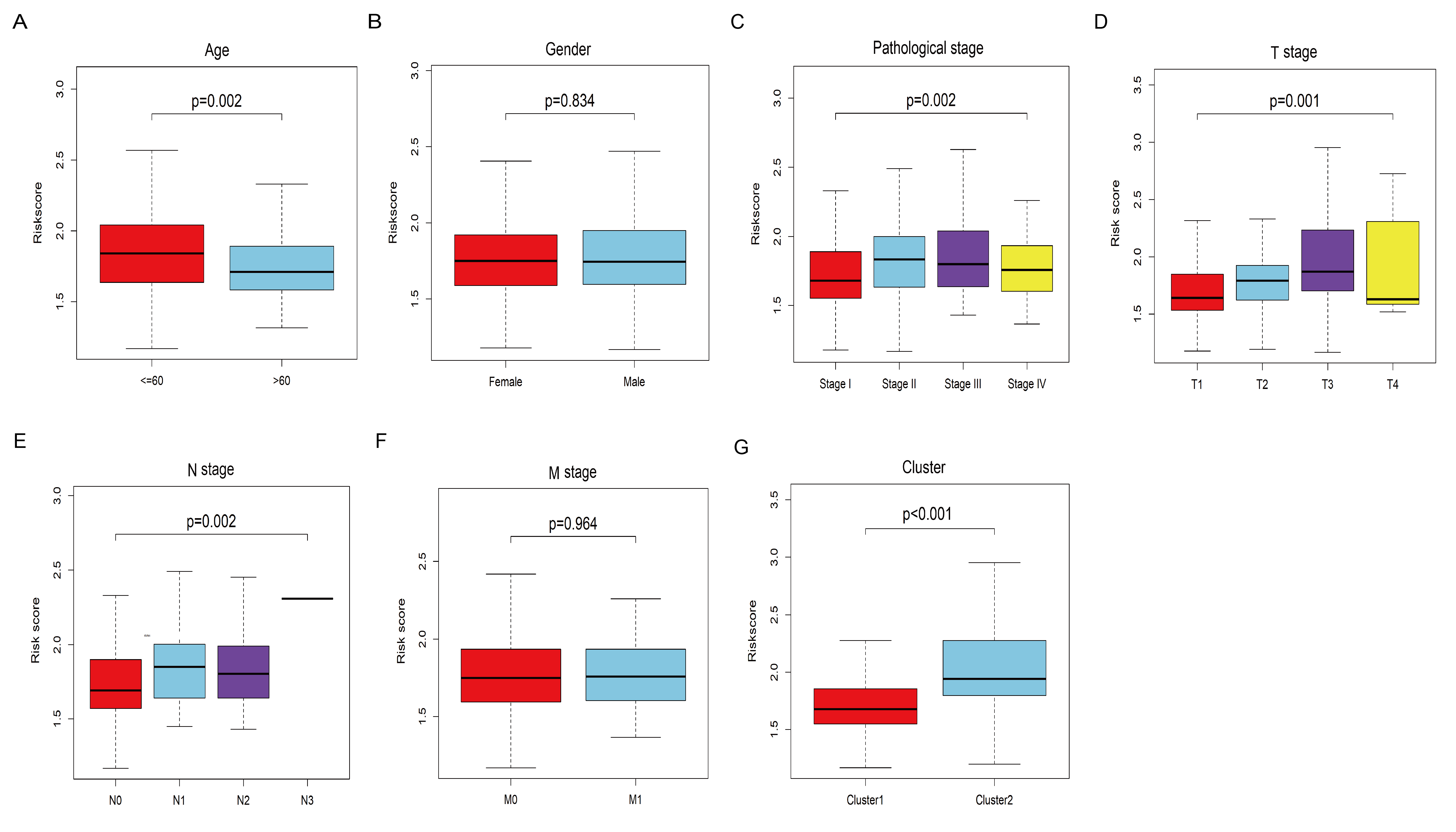

Supplement: Supplementary Figure 1 — Association between the risk score, clinicopathological features, and cluster 1/2 subgroups in TCGA dataset. Distribution of risk scores in LUAD patients stratified by age (A), gender (B), pathological stage (C), T stage (D), N stage (E), M stage (F) and cluster 1/2 subgroup (G). [file Image_1.tiff]
