# Supplementary material for: Ceramide Pathway Regulators Predict Clinical Prognostic Risk and Affect the Tumor Immune Microenvironment in Lung Adenocarcinoma
Source: Front Oncol. 2020 Oct 27;10:562574. doi: 10.3389/fonc.2020.562574 (PMC7653182; doi:10.3389/fonc.2020.562574)
Supplement: Supplementary Table 1 — Gene list of ceramide pathway. [file Table_1.docx]

**Supplementary Table S1. Gene list of ceramide pathway.**

| **Entrez Gene Id** | **Symbol** | **Name** |
| --- | --- | --- |
| 9131 | AIFM1 | Apoptosis-inducing factor, mitochondrion-associated, 1 |
| 572 | BAD | BCL2-associated agonist of cell death |
| 581 | BAX | BCL2-associated X protein |
| 596 | BCL2 | B-cell CLL/lymphoma 2 |
| 841 | CASP8 | Caspase 8, apoptosis-related cysteine peptidase |
| 54205 | CYCS | Cytochrome c, somatic |
| 8772 | FADD | Fas (TNFRSF6)-associated via death domain |
| 5604 | MAP2K1 | Mitogen-activated protein kinase kinase 1 |
| 6416 | MAP2K4 | Mitogen-activated protein kinase kinase 4 |
| 4214 | MAP3K1 | Mitogen-activated protein kinase kinase kinase 1, E3 ubiquitin protein ligase |
| 5594 | MAPK1 | Mitogen-activated protein kinase 1 |
| 5595 | MAPK3 | Mitogen-activated protein kinase 3 |
| 5599 | MAPK8 | Mitogen-activated protein kinase 8 |
| 4790 | NFKB1 | Nuclear factor of kappa light polypeptide gene enhancer in B-cells 1 |
| 8439 | NSMAF | Neutral sphingomyelinase (N-SMase) activation associated factor |
| 5894 | RAF1 | V-raf-1 murine leukemia viral oncogene homolog 1 |
| 5970 | RELA | V-rel reticuloendotheliosis viral oncogene homolog A (avian) |
| 8737 | RIPK1 | Receptor (TNFRSF)-interacting serine-threonine kinase 1 |
| 6609 | SMPD1 | Sphingomyelin phosphodiesterase 1, acid lysosomal |
| 7132 | TNFRSF1A | Tumor necrosis factor receptor superfamily, member 1A |
| 8717 | TRADD | TNFRSF1A-associated via death domain |
| 7186 | TRAF2 | TNF receptor-associated factor 2 |
